# A transcriptomics model of estrogen action in the ovine fetal hypothalamus: evidence for estrogenic effects of ICI 182,780

**DOI:** 10.14814/phy2.13871

**Published:** 2018-09-16

**Authors:** Maria Belen Rabaglino, Maureen Keller‐Wood, Charles E. Wood

**Affiliations:** ^1^ Department of Physiology and Functional Genomics College of Medicine University of Florida Gainesville Florida USA; ^2^ Pharmacodynamics College of Pharmacy University of Florida Gainesville Florida USA

**Keywords:** Brain development, estrogen signaling pathway, fetal programming, microarray

## Abstract

Estradiol plays a critical role in stimulating the fetal hypothalamus–pituitary–adrenal axis at the end of gestation. Estradiol action is mediated through nuclear and membrane receptors that can be modulated by ICI 182,780, a pure antiestrogen compound. The objective of this study was to evaluate the transcriptomic profile of estradiol and ICI 182,780, testing the hypothesis that ICI 182,780 antagonizes the action of estradiol in the fetal hypothalamus. Chronically catheterized ovine fetuses were infused for 48 h with: vehicle (Control, *n* = 6), 17*β*‐estradiol 500 *μ*g/kg/day (Estradiol, *n* = 4), ICI 182,780 5 *μ*g/kg/day (ICI 5 *μ*g, *n* = 4) and ICI 182,780 5 mg/kg/day (ICI 5 mg, *n* = 5). Fetal hypothalami were collected afterward, and gene expression was measured through microarray. Statistical analysis of transcriptomic data was performed with Bioconductor‐R and Cytoscape software. Unexpectedly, 35% and 15.5% of the upregulated differentially expressed genes (DEG) by Estradiol significantly overlapped (*P* < 0.05) with upregulated DEG by ICI 5 mg and ICI 5 *μ*g, respectively. For the downregulated DEG, these percentages were 29.9% and 15.5%, respectively. There was almost no overlap for DEG following opposite directions between Estradiol and ICI ICI 5 mg or ICI 5 *μ*g. Furthermore, most of the genes in the estrogen signaling pathway – after activation of the epidermal growth factor receptor – followed the same direction in Estradiol, ICI 5 *μ*g or ICI 5 mg compared to Control. In conclusion, estradiol and ICI 182,780 have estrogenic genomic effects in the developing brain, suggesting the possibility that the major action of estradiol on the fetal hypothalamus involves another receptor system rather than estrogen receptors.

## Introduction

Preparation of the fetus for birth involves the interaction of several endocrine systems. Estrogens, secreted by the placenta, influence fetal hypothalamic function and in turn, affect the timing of birth and fetal readiness for life outside the uterus. In ruminants, for example, initiation of parturition is a consequence of the neuroendocrine cascade between the components of the hypothalamus‐pituitary‐adrenal (HPA) axis (Liggins et al. 1973). Treatment of the fetus with physiologically relevant levels of estradiol or estradiol sulfate increases circulating fetal ACTH and cortisol concentrations (Saoud and Wood 1997; Winikor et al. 2011; Wood 2011). We have assumed that the increase in fetal HPA axis activity was mediated by estradiol binding to the estrogen receptor or, in the case of estradiol sulfate, deconjugation of estradiol sulfate preceding the binding of estradiol with the estrogen receptor (Wood 2011).

ICI 182,780 is a selective estrogen antagonist that has been shown to interfere with the nucleocytoplasmic shuttling of the estrogen receptor (ER) by blocking the nuclear uptake of the receptor (Dauvois et al. 1993). This drug has been a useful tool in basic science experiments for assessing estrogen receptor‐mediated actions of estrogens (Wakeling and Bowler 1992; Howell et al. 2000). Clinically, it is used in the treatment of breast cancer (among other applications), since it is an efficacious antagonist of the proliferative actions of estrogen ‐ER dependent‐ in reproductive organs such as the breast and uterus (Howell et al. 2000). In previous experiments, we used ICI 182,780 in in vivo experiments designed to test the effect of estrogen receptor blockade on mRNA expression of CRH, propiomelanocortin (POMC) (Wood et al. 2001; Schaub et al. 2008b), ER (Schaub and Wood 2009) and prostaglandin synthase‐2 (PGHS2) (Schaub et al. 2008b), which are genes involved in the control of ACTH secretion (Schaub et al. 2008b). These studies were based in the assumption that estradiol action was primarily via classical ER alpha (ESR1) and beta (ESR2) and that ICI 182,780 worked in vivo purely as an ER antagonist. However, the overall effect of blocking estrogen receptors in the transcriptomic of fetal hypothalamus close to parturition – when the HPA axis activity is increasing – is still unknown.

The objective of this study was to evaluate the fetal hypothalamic transcriptomic response to intravenous infusions of estradiol and ICI 182,780 at different doses during 2 days in late gestation, testing the hypothesis that ICI 182,780 has an action opposite to that of estradiol in the fetal hypothalamus. We used an Agilent microarray of ovine transcripts that we annotated, assigned human gene names, and have used for other studies to investigate the mechanism of responses to various stimuli, including fetal hypoxia (Wood et al. 2013, 2016; Chang et al. 2016) and cerebral hypoperfusion (Wood et al. 2014). Array annotation has been validated in these and other studies.

## Materials and Methods

### Animal procedures

All these experiments were performed in accordance with the Guiding Principles for Research Involving Animals and Human Beings published by the American Physiological Society and were approved by the University of Florida Institutional Animal Care and Use Committee. A total of 11 pregnant ewes were used in this study. Three ewes were carrying singletons, and the other 8 were pregnant with twin fetuses. The gestational age at the time of surgery was 124–126 days of gestation (term: ~145 days of gestation). The fetuses were randomly assigned to the groups at the time of surgery. If the fetuses were twins, both animals were assigned to the same treatment group. Three sets of twin fetuses (*n* = 6 total) were infused with DMSO:water::50:50 vehicle solution (*n* = 4) or were catheterized but did not receive infusions (*n* = 2; Control group), two sets of twins were administered 17*β*‐estradiol 500 *μ*g/kg/day infusion (Estradiol, *n* = 4); another two sets were administered ICI 182,780 5 *μ*g/kg/day infusion (ICI 5 *μ*g, *n* = 4) and three singletons and one set of twin were treated with ICI 182,780 5 mg/kg/day infusion (ICI 5 mg, *n* = 5). For all experiments, estradiol was purchased from Sigma Chemical (catalog number E8875, St. Louis, MO), ICI 182,780 was purchased from Tocris Biosciences (catalog number 1047, Bristol, UK), and DMSO was purchased from Sigma Chemical. The estradiol dose (500 *μ*g/kg/day infusion) is known to produce physiological increases in fetal plasma estradiol concentration, and the dose of ICI 182,780 was scaled to the estradiol dose (5 *μ*g/kg/day infusion). However, to avoid bias on the action of ICI 182,780 due to the increasing number of estrogen receptors in the developing brain, we arbitrarily increased the dose 1000 times to 5 mg/kg/day infusion. All animals were housed in individual pens located in the Animal Resources Department at the University of Florida. The rooms maintained controlled lighting and temperature, and sheep were given food and water ad libitum.

Food was withheld from the pregnant ewes for 24 h before surgery. Ewes were intubated and anesthetized with halothane (0.5–2%) in oxygen before and during surgical preparation as previously described (Wood et al. 2003). Surgery and catheter placement for all fetuses were performed using an aseptic technique as previously described, with femoral arterial, and venous catheters as well as amniotic fluid catheters (Wood et al. 2003; Schaub et al. 2008b). Vascular catheters were exteriorized through the flank of the ewe using a trochar, where they were maintained in a removable synthetic cloth pocket. Ewes were treated with 1 mg/kg flunixin meglumine (Webster Veterinary, Sterling, MA) for analgesia and returned to their pens where they were monitored until they could stand on their own. If needed, a second treatment with flunixin meglumine was administered 24–48 h after the first treatment with this drug. Twice daily during a 5‐day recovery period, ewes were treated with an antibiotic (ampicillin, 750 mg *im*: Polyflex^®^, Fort Dodge Laboratories, Fort Dodge, IA) or Cefazolin (11–22 mg/kg, iv or im) and rectal temperatures were monitored for indication of postoperative infection. None of the animals included in this study showed any signs of postoperative infection.

### Experimental procedure and blood sample collection

After the 5‐day recovery period, compounds (estradiol, ICI 5 *μ*g/kg, ICI 5 mg/kg and vehicle) were infused into the fetuses intravenously. Infusions were continued for 48 h, allowing enough time for genomic actions after alteration of steroid action. The infusions were delivered with an SAI MiniBT Infusion pump (Strategic Applications Inc., Chicago, IL), which was calibrated to dispense the 10 mL continuously and uniformly during the 48 h treatment period.

### Tissue sample collection

Pregnant ewes and fetuses were euthanized with an overdose of sodium pentobarbital after the 48 h of the treatment period. Brains were rapidly removed, dissected into distinct regions, and snap frozen in liquid nitrogen. Tissues were collected from brain regions and selected organs and archived for other experiments. In the present microarray experiment, we analyzed gene expression in the hypothalamus because of its critical role in endocrine control mechanisms, including fetal stress responses and timing of birth in the sheep (McDonald and Nathanielsz 1991). Dissection of the hypothalamus from the fetal brain was performed as described in Gersting et al. (2009) and in Schaub et al. (2008a). Hypothalamus was removed as a single block of tissue, boundedon the rostral edge by the rostral edge of the optic chiasm, on the caudal side by the caudal edge of the median eminence, and on the sides by the edges of the median eminence.

### RNA extraction and preparation

RNA was extracted from the entire hypothalamus using Trizol (Invitrogen, Carlsbad, CA) following the manufacturer's directions. The RNA was resuspended in RNAsecure, and stored at −80°C in aliquots until use. For microarray analysis, 60 *μ*g of these RNAs were DNase treated using the Turbo RNase‐free DNase kit (Ambion, Foster City, CA), the concentration determined with a Nanodrop spectrophotometer (ND‐1000, ThermoFisher, Wilmington, DE) and the integrity of the RNA was measured using an Agilent Bioanalyzer, 2100 model. The RNA Integrity Number (RIN) value for the RNAs ranged from 7.1 to 8.6. One microgram of the DNase‐treated RNA was labeled with Cyanine 3 (Cy3) CTP with the Agilent Quick Amp kit (5190‐0442, New Castle, DE) according to their methodology, purified with the Qiagen RNeasy kit (Valencia, CA) according to Agilent's revision of the Qiagen protocol as shown in the Quick Amp kit protocol except that the microcentrifugation spins were performed at room temperature instead of 4°C. The resulting labeled cRNA was analyzed with the NanoDrop spectrophotometer, and the specific activities and the yields of the cRNAs were calculated; these ranged from 10.22 to 12.38 pmol Cy3/*μ*g RNA and from 5.6 to 8.9 *μ*g, respectively. The labeled cRNA was stored at −80°C until use.

### Microarray hybridization

This was performed following protocols from Agilent. Briefly, 600 ng of each labeled cRNA was fragmented and then mixed with hybridization buffer using the Agilent gene expression hybridization kit. These were applied to an ovine 8 X 15 K array slide (Agilent 019921), containing 8 arrays with 15,208 oligomers with a length of 60 bases and hybridized at 65°C for 17 h at 10 rpm. The arrays were washed, dried, stabilized, and scanned with an Agilent G2505B 2 dye scanner at the Interdisciplinary Center for Biotechnology Research at the University of Florida. Features were extracted with Agilent Feature extraction 9.1 software. Microarray data have been deposited in the NCBI Gene Expression Omnibus under accession number GSE99497.

### Statistical analysis

The limma package was used to import the raw data into R (http://www.r-project.org), perform background correction and normalize the data using the quantile normalization method (Smyth 2005). Control probes and low expressed probes were filtered out, retaining for further analysis the probes that were at least 10% brighter than the negative controls on at least four arrays. Batch effects between slides were removed with the ComBat function of the SVA package (Johnson et al. 2007). Then, the limma package was used for the statistical analysis, applying the empirical Bayes method proposed by Smyth (Smyth 2004). This method calculates a moderated *t*‐statistic for differential expression for each gene by performing a linear model fit on the data. An empirical Bayes step is applied to moderate the standard errors of the estimated log‐fold changes and produce more stable estimates, especially when the number of replicates is small.

Each treatment group was compared with the control group. A gene was considered to be significantly differentially expressed (over or under expressed) if both of the following conditions were met: (1) the ratio of the normalized intensity in the treatment fetus sample to normalized intensity in the control fetus sample was higher or lower than a twofold change (up or downregulation, respectively); and (2) differences were considered statistically significant at *P* ≤ 0.05.

### Data comparison

Up and downregulated differentially expressed genes (DEG) by each of the treatments were compared to each other to determine the overlap of DEG between them. Statistical comparisons were made by the test independence (Pearson's chi‐square test) to determine the relatedness between up or downregulated DEG by estradiol and up or downregulated DEG by ICI 5 μg or ICI 5 mg.

### Clustering analysis

The network inference and clustering analysis were performed using Cytoscape version 3.6.0 (Smoot et al. 2011), through the following plugins: GeneMania, ClusterONE, and BINGO. GeneMania was used to infer networks of functionally related genes (Warde‐Farley et al. 2010) using known functional associations from the Homo sapiens database. The list of human official symbols for the genes of interest was input into the GeneMania plugin to retrieve the corresponding association network. The network was inferred for the upregulated and downregulated genes for each of the treatments compared to the control.

These networks were merged using the Merge plugin, included in the Cytoscape core, in order to detect overlapped genes between networks. Four types of merges were performed (1) between the networks composed of upregulated genes for each treatment; (2) between networks composed of downregulated genes for each treatment, (3) between networks inferred with upregulated genes for the estradiol treatment and downregulated genes for ICI (both doses) treatments and (4) between networks containing downregulated genes for the estradiol treatment and upregulated genes for ICI (both doses) treatments.

ClusterONE was used to discover densely connected and possibly overlapping regions (clusters) within subnetworks (Nepusz et al. 2012). A *P*‐value is calculated for each cluster, based on the one‐sided Mann–Whitney U test performed on the in‐weights and out‐weights of the vertices. An in‐weights value significantly larger than the out‐weights value would indicate a valid cluster and not the result of random fluctuations. Thus, a *P*‐value is assigned to the cluster. Only the clusters with a *P*‐value less than 0.05 were considered in further analyses.

BiNGO was run to determine which biological processes are statistically overrepresented in the set of genes corresponding to the identified cluster (Maere et al. 2005). The statistical test employed was the hypergeometric test (equivalent to the Fisher test). The threshold *P*‐value was 0.05, after correction by the Bonferroni method.

### Differential expression testing for genes in a KEGG pathway network

Despite the overrepresented biological processes identified with BiNGO (see above), it was expected that genes in the estrogen signaling pathway would follow opposite directions in Estradiol and ICI 5 *μ*g or ICI 5 mg compared with the control group. To prove this hypothesis, the KEGG pathway “map04915” (estrogen signaling pathway) was first translated into a gene network with the KEGGraph package (Zhang and Wiemann 2009). Then, the DEGraph package (Jacob et al. 2012) was used to test whether this gene network was differentially expressed between Estradiol, ICI 5 *μ*g or ICI 5 mg and the control group. As result, significant subnetworks composed by nodes that are colored according to the *t*‐statistics (or *t*‐score) for the mean difference of expression between the two conditions for each gene were obtained. This method takes into account the topology of the network to yield more powerful detection procedures than the classical two‐step approaches, which first test individual genes, then test gene sets for enrichment in DEG.

## Results

**Figure 1:**
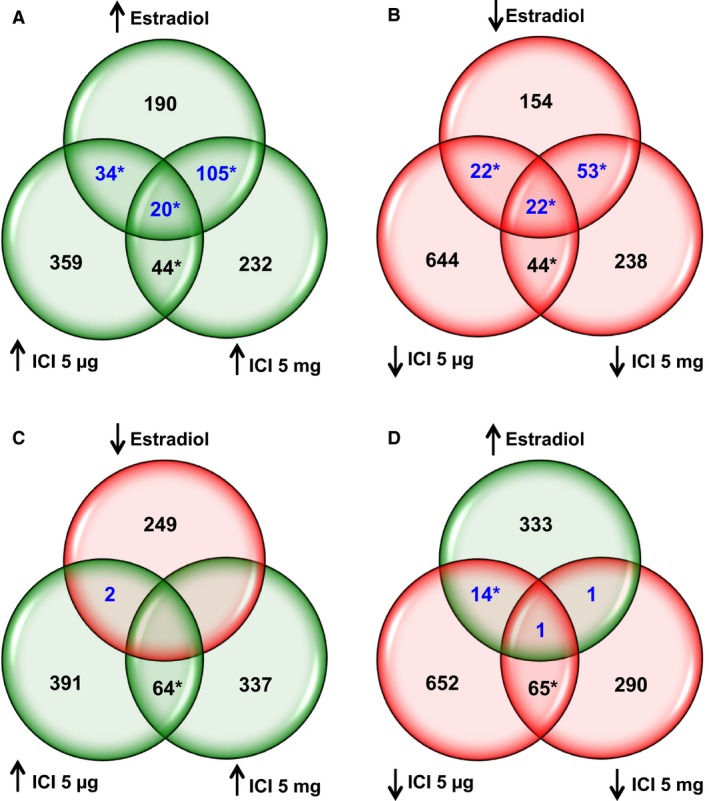
Venn diagrams showing the overlap for the differentially expressed genes (DEG) in fetal hypothalami after 48 h treatment with 17*β*‐estradiol (500 *μ*g/kg/day; Estradiol, *n* = 4), low dose of ICI 182,780 (5 *μ*g/kg/day; ICI 5 *μ*g, *n* = 4) or high dose of ICI 182,780 (5 mg/kg/day; ICI 5 mg, *n* = 5), compared to controls (*n* = 6). (A) Upregulated DEG by all the treatments. (B) Downregulated DEG by all the treatments. (C) Downregulated DEG by Estradiol and upregulated DEG by both doses of ICI 182,780. (D) Upregulated DEG by Estradiol and downregulated DEG by both doses of ICI 182,780. (*P < 0.05 by Pearson chi‐square test).

In the fetal hypothalamus, intravenous infusion of estradiol upregulated 349 genes and downregulated 251 genes. In comparison, ICI 182,780 treatment upregulated 457 and downregulated 378 genes when administered in a dose of 5 *μ*g/kg, and upregulated 401 and downregulated 358 genes when administered in a dose of 5 mg/kg. Surprisingly, there was significant overlap between differentially expressed genes (DEG) altered in the same direction (up or downregulated DEG, Fig. 1A and B, respectively) but no significant overlap for genes altered in the opposite direction (Fig. 1C and D) by Estradiol and ICI (5 *μ*g or ICI 5 mg). These results suggested a similar transcriptomic response in the fetal hypothalamus after ICI 182, 780 or estradiol infusions for 48 h.

To confirm this finding, up and downregulated DEG were organized into inferred networks and visualized with the Cytoscape software. The nodes (i.e., DEG) of the networks were related to each other if they accomplished at least one of the following criteria: to belong to the same pathway, to be coexpressed, to present physical (protein–protein) or genetic interactions, to be colocalized, to share protein domains, or to be predicted to interact in other organisms. The number of nodes in the networks inferred with the upregulated DEG was 344 for the estradiol treatment, 388 for the ICI 5 mg treatment and 438 for the ICI 5 *μ*g treatment. For the downregulated DEG, the number of nodes in each network was 242 for the estradiol treatment, 343 for the ICI 5 mg treatment, and 367 for the ICI 5 *μ*g treatment.

**Figure 2:**
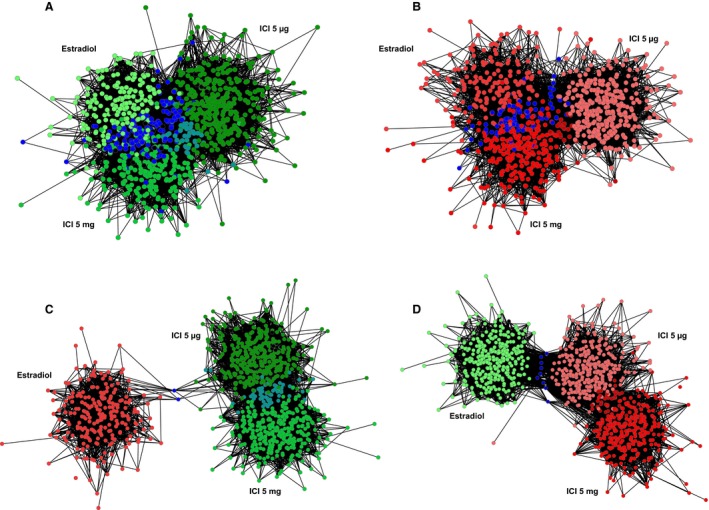
Merged networks of functionally related differentially expressed genes (DEG) in fetal hypothalami after 48 h treatment with 17*β*‐estradiol (500 *μ*g/kg/day; Estradiol, *n* = 4), low dose of ICI 182,780 (5 *μ*g/kg/day; ICI 5 *μ*g, *n* = 4) or high dose of ICI 182,780 (5 mg/kg/day; ICI 5 mg, *n* = 5), compared to controls (*n* = 6). (A) Upregulated DEG by all the treatments. (B) Downregulated DEG by all the treatments. (C) Downregulated DEG by Estradiol and upregulated DEG by both doses of ICI 182,780. (D) Upregulated DEG by Estradiol and downregulated DEG by both doses of ICI 182,780. Blue nodes denote the overlapped DEG between the networks.

These networks were merged to visualize the overlapping nodes between them (Fig. 2). In concordance with the Venn Diagrams shown in Figure 1, there was a strong overlap between networks of upregulated DEG for each of the three treatments (Fig. 2A) as well as between networks of downregulated DEG for each of the three treatments (Fig. 2B). The overlap was even more evident between the networks of DEG in the estradiol group and ICI 5 mg group. However, there was little overlap between networks of DEG following opposite directions in the estradiol group as compared to either the ICI 5 mg group or ICI 5 *μ*g group (Fig. 2C and D). Thus, the transcriptomic effects for the treatment with both doses of ICI 182, 780 in the fetal hypothalamus are rather similar to the transcriptomic effects of estradiol, at least after 48 h of infusion. Next, in order to determine the functional significance of these findings, we interrogated the merged networks displayed in Figures 2A and B for clusters of genes in the overlapping and nonoverlapping regions of the merged network. Statistically overrepresented biological processes for these clusters are summarized in Table 1A and B, respectively (redundant biological processes were simplified).

**Table 1:** Statistically over‐represented biological processes enriched by upregulated (A) or downregulated (B) differentially expressed genes (DEG) in ovine fetal hypothalami after 48 h treatment with 17*β*‐estradiol (500 *μ*g/kg/day, *n* = 4), low (5 *μ*g/kg/day, *n* = 4) or high (5 mg/kg/day, *n* = 5) dose of ICI 182,780, compared to controls (*n* = 6)

Subnetwork	Overrepresented biological processes		Genes
(A) Networks containing upregulated DEG for all treatments			
Overlap	Regulation of cell migration/locomotion		CDH13‐LAMA4‐PTPRM‐TIE1‐JAG1‐IGFBP3‐VCL
	Angiogenesis		CDH13‐LAMA4‐COL4A1‐LEPR‐VEGFA‐FOXC1‐TIE1‐JAG1‐VEZF1‐CDH5
	Cell adhesion		CDH13‐LAMA4‐STAT5B‐NID1
	Cell differentiation/development		NBN‐HTATIP2‐CADM1‐STAT5B‐NNAT‐JAG1‐ACAT1‐SLC7A5‐CDH4‐PCSK1‐PEG10‐SLC1A3‐PBXIP1‐TEAD4‐MSI2‐BCL6‐PATZ1‐TIE1‐VEZF1‐SATB1‐COL4A1‐PTPRM‐PLXNB2‐LEF1‐WWTR1‐NOTCH2‐LAMA4‐VEGFA‐FOXC1‐GGNBP2
	Myeloid cell differentiation		L3MBTL1‐STAT5B‐GNAS‐RARA‐JAG1
Not‐overlap estrogen	Glucose metabolic process		GPI‐LDHA‐PGAM2‐ENO1
	Response to external stimulus		ALPL‐LDHA‐AXL‐ADNP‐USF1‐PTEN‐SLIT2‐CXCL10‐TGFB2‐ASGR1‐PITPNM1‐NBR1‐PDGFRB‐BMP7‐CTSH‐CLOCK‐ACSL5
Not‐overlap ICI 5 *μ*g/kg	Positive regulation of synaptic transmission		CCL2‐IFNG‐OXTR‐GRIA4
	Response to stress		CCL2‐EDN1‐FOS‐CTGF‐IFNG‐THBS1‐PTX3‐FGF2‐ANGPTL4‐MAFF‐SELP‐HERPUD1‐CEBPB‐FLT1‐IL8‐SOCS3‐SMAD7‐NR4A2‐DDIT4‐S100A12‐THBD‐ADM‐BTG2‐DUSP1‐JUN‐DNAJB1‐CD14
	Response to oxygen levels		FLT1‐CCL2‐ADM‐SOCS3‐CTGF‐EDN1‐NR4A2‐THBS1‐ANGPTL4‐DDIT4
	Angiogenesis		SELP‐FLT1‐IL8‐SMAD7‐SOCS3‐EDN1‐TIPARP‐FOXO1‐JUNB‐JMJD6‐CTGF‐JUN‐THBS1‐FGF2‐CYR61
	Response to progesterone stimulus		FOS‐CCL2‐SOCS3‐THBS1‐JUNB
	Phosphoinositide 3‐kinase cascade		IGF1R‐EDN1‐PIK3R1
Not‐overlap ICI 5 mg/kg	No significant over‐represented biological processes at corrected *P*‐value < 0.05		
(B) Networks containing downregulated DEG for all treatments			
Overlap		Oxidative phosphorylation	FXN‐ATP5B‐ATP5C1‐ATP5G1‐NDUFS3‐ATP5H
		Mitochondrial transport	SLC25A20‐ATP5B‐TIMM17B‐ATP5C1‐ATP5H
		Energy coupled proton transport, down electrochemical gradient	ATP5B‐ATP5C1‐ATP5G1‐ATP5H
Not‐overlap estrogen		Oxidative phosphorylation	ATP5F1‐ATP5A1‐NDUFS1
		Oxidation reduction	GPD1‐PECR‐PHYH‐BDH1‐NDUFS1‐ETFA
		ATP metabolic process	ATP5S‐ATP5F1‐ATP5A1‐NDUFS1
Not‐overlap ICI 5 *μ*g/kg		RNA splicing	RALY‐FUS‐POLR2E‐RNPS1‐SF3A2‐DDX5‐XAB2‐SART1‐PRPF6‐SF3B2‐DHX38‐DDX23‐PRPF8‐THOC6‐GEMIN6‐RBM10‐CPSF3‐PUF60‐CPSF1
		Cell cycle	CDC23‐HMG20B‐ILF3‐MBD3‐SART1‐GPS2‐NCAPH‐MCM7‐GSPT1‐POLD1‐CHTF18‐PSMD3‐DYNC1H1‐SUPT5H
		Mitotic cell cycle	NCAPH‐FZR1‐GSPT1‐POLD1‐KATNB1‐CDC23‐PSMD3‐DYNC1H1
Not‐overlap ICI 5 mg/kg		Electron transport chain	CYB561D2‐UQCR10‐UQCR11‐UQCRC1‐TXN2‐NDUFA13
		Mitochondrial ATP synthesis coupled electron transport	UQCR10‐UQCR11‐UQCRC1
		Oxidative phosphorylation	UQCR10‐UQCR11‐UQCRC1‐ATP6V0B
		Oxidation reduction	CYB561D2‐HSD17B10‐UQCR10‐UQCR11‐UQCRC1‐TXN2‐NDUFA13‐SCO2‐ALKBH4

**Figure 3:**
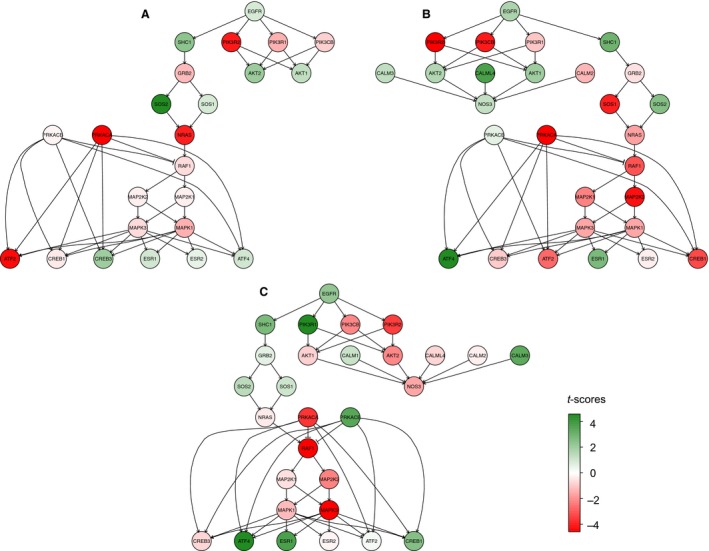
Subnetworks obtained after testing the “estrogen signaling pathway” network (KEGG pathway: map04915) in differentially expressed genes in fetal hypothalami after 48 h of treatment with (A) 500 *μ*g/kg/day of 17*β*‐estradiol (*n* = 4); (B) 5 mg/kg/day of ICI 182,780 (*n* = 5); and (C) 5 *μ*g/kg/day of ICI 182,780 (*n* = 4); compared to controls (*n* = 6). T‐score refers to the t‐statistics obtained after applying individual *t*‐test to each gene comparing both conditions (treatments vs. control).

Despite the overrepresented biological processes summarized in Table 1, we explored the question of whether genes part of the known estrogen signaling pathway would follow opposite directions in Estradiol and ICI 5 *μ*g or ICI 5 mg compared to the control group. Using a pathway‐driven approach, we tested the KEGG pathway 0491: “estrogen signaling pathway”, translated as a network, in Estradiol, ICI 5 mg or ICI 5 *μ*g, versus the control group. The resulting significant subnetworks (Fig. 3) showed the same signaling pathway for all groups compared with the controls: activation of the epidermal growth factor receptor (EGFR), which is a member of a membrane‐initiated steroid‐signaling pathway (Filardo 2002). Most of the downstream nodes on the pathway changed the same direction in Estradiol, ICI 5 mg or ICI 5 *μ*g compared to the control group. The subnetworks for Estradiol and ICI 5 mg (Fig. 3A and B, respectively) contained these similarly upregulated nodes: EGFR, AKT1, AKT2, SHC1, SOS2, ESR1, and ATF4; and the following downregulated nodes: PIK3R1, PIK3R2, PIK3CB, GRB2, NRAS, RAF1, MAP2K1, MAP2K2, MAPK1, MAPK3, PRKACA, CREB1, and ATF2. Only PRKACB, SOS1, ESR2, and CREB3 were changed in opposite directions. The subnetworks for Estradiol and ICI 5 *μ*g (Fig. 3A and 3C, respectively) contained the following upregulated nodes in common: EGFR, SHC1, SOS1, SOS2, ESR1, and ATF4; and the following downregulated nodes: PIK3R2, PIK3CB, NRAS, RAF1, MAP2K1, MAP2K2, MAPK1, MAPK3, and PRKACA. The nodes with opposite directions were GRB2, AKT1, AKT2, PIK3R1, PRKACB, ESR2, CREB1, and CREB3.

As mentioned above, most of the nodes in the signaling pathway followed the same direction in Estradiol, ICI 5 *μ*g or ICI 5 mg groups compared with the control group. The direction of the genes corresponding to factors found within the cytoplasm could vary relative to the dynamics of the responses to the different ligands since it has been shown that recruitment of the downstream signaling proteins differs between EGFR agonists and also between different doses of the same agonist (Ronan et al. 2016). However, it is interesting to note that the membrane receptor (EGFR) and one of the most important nuclear ligand‐activated transcription factors for estradiol (ESR1) are upregulated in all the treatments, although the difference of expression for these genes between the treated and control fetuses in the general statistical analysis was not significant.

These findings reinforce the concept that, after 48 h of infusion, ICI 182,780 exerts an estrogenic effect in the ovine fetal hypothalamus.

## Discussion

This study demonstrates that the transcriptomic response to a 2 days infusion of ICI 182,780 is similar to, not opposite to, the transcriptomic response to a 2 days infusion of estradiol (Figs. 1 and 2, panels A–B). The concordance of differential gene expression patterns in the Estradiol and ICI 182,780 treatment groups resulted in an overlap of biological processes (Tables 1). Also, Estradiol and ICI 182,780 treatments stimulate the estrogen‐signaling pathway in a similar fashion (Fig. 3). ESR1 was shown to be upregulated by all the treatments, although the difference was not statistically significant. In the rat brain, estrogen exposure downregulated ESR1 (Brown et al. 1996). In the ovine fetal hypothalamus, estradiol treatment did not modify the mRNA expression of ESR1 but downregulated its protein levels (Wood 2008). To determine the physiological changes in ESR1 and ESR2 mRNA ontogenies, we re‐analyzed the microarray results from ovine fetal hypothalamic samples obtained at 80, 100, 120, 130, 145 days of gestation and 1 day of extrauterine life (Rabaglino et al. 2014). We found that ESR1 mRNA expression followed an increasing trend, being significantly higher at 145 days of gestation compared to the other gestational ages but decreased at 1 day of life. ESR2 mRNA expression remained mostly unchanged (Fig. S1). In order to corroborate these microarray results, mRNA levels were also determined by qRT‐PCR. ESR1 mRNA expression followed the increasing trend, although the higher levels were observed at 1 day of life, to decrease at 1 week of life. ESR2 expression remained unchanged (Fig. S2, A–B). Circulating estrogen levels in the ovine fetus increase toward the end of gestation, playing a critical role in brain maturation and stimulation of the HPA axis for parturition (Wood 1999, 2005, 2014). Thus, the action of exogenous estradiol treatment on ESR1 expression may depend on the time of gestation and level of maturation of the fetal brain.

There is ample evidence that ICI 182,780 interacts directly with the estrogen receptor. ICI 182,780 is a structural analog of 17*β* estradiol and binds ESR1 and ESR2 with similar affinity compared to that of 17*β* estradiol. The mechanism of action of ICI 182,780 is associated with a conformational change at the estrogen receptor, which in turn seemingly impairs the dimerization (Brzozowski et al. 1997) and nuclear uptake of the receptor, both necessary steps for binding the receptor to its DNA binding site (Dauvois et al. 1993). In addition, other mechanisms specific to mammalian cells can also trigger an antiestrogen activity of ICI 182,780, including compound‐receptor binding caused by different cytoplasmic components or disruption of the cytoplasm‐to‐nuclear transport systems (Dudley et al. 2000).

However, ICI 182,780 has been shown to have agonist activity in some systems. In yeast, which lack endogenous nuclear receptors and co‐regulatory proteins of other receptors, (Walfish et al. 1997), ICI 182,780 induced dimerization and transcriptional activity of transfected ER and so it did not antagonize the effect of estradiol (Dudley et al. 2000). In fish, ICI 182, 780 induced an agonist response to estradiol at the level of gene expression in liver but not in testis (Pinto et al. 2006). Thus, ICI 182,780 is known to be a selective ER modulator, but its mechanism of action can differ among the species, cell type, and probably other variables as well. Indeed, for mammalian cells, the effect of ICI 182,780 has been shown not to be exclusively estrogen antagonist, at least in tissues other than adult reproductive tissues such as breast and uterus. Interestingly, agonist effects of ICI 182,780 have been observed in the brain in several studies in vitro. In one study, ICI 182,780 activated neuronal responses similar to 17*β* estradiol to induce neuronal plasticity and neuroprotection in fetal rat hippocampal neurons (Zhao et al. 2006). Also, 17*β* estradiol and ICI 182,780 were shown to activate ERK1/2 MAPK signaling in developing and mature cerebellar neurons, and the continuous exposure to estradiol had an antimitotic effect (Wong et al. 2003). Another study demonstrated that 17*β* estradiol stimulated axon growth in hypothalamic neurons in vitro, and this effect was not blocked with ICI 182,780 (Cambiasso and Carrer 2001). Therefore, these in vitro studies demonstrated a similar effect for both 17*β* estradiol and ICI 182,780 in the developing brain.

In this study, the upregulated DEG by all treatments were significantly enriched for biological processes such as angiogenesis and cell migration, which are critical during fetal development (Table 1A). Estrogen has shown to induce angiogenesis in brain tissues (Krause et al. 2006) or to stimulate actin cytoskeleton remodeling, cell adhesion and cell migration (Giretti and Simoncini 2008). Our results are consistent with these actions, and they suggest that ICI 182,780 is rather an agonist than an antagonist of estradiol regarding these actions. The overrepresented biological processes enriched with the downregulated DEG by all the treatments were mostly related to the mitochondrial respiratory chain (Table 1B). Estradiol has complex actions on the respiratory chain. Several works have demonstrated that 17*β* estradiol enhances mitochondrial efficiency and induces the transcription of mitochondrial respiratory chain protein, as reviewed by Chen et al. (2009). However, 17*β* estradiol decreased mitochondrial respiratory activity and oxidative phosphorylation in liver cells, and these effects were not suppressed with ICI 182,780 (Moreira et al. 2006). In addition, 17*β* estradiol inhibited mitochondrial electron transport in homogenates of rat uterus, liver, and skeletal muscle (Kuss and Jutting 1967). Therefore, the action of 17*β* estradiol on the respiratory chain depends upon the context, tissue type (Moreira et al. 2011), and age (Alvarez‐Delgado et al. 2010). Downregulation of genes related to the cell cycle occurs physiologically in the ovine fetal brain during the last stage of gestation (Rabaglino et al. 2014). It is possible that physiologically, circulating estradiol decreases the mitochondrial respiratory function as part of the adaptive mechanism that balances the increased oxygen demand with the limited supply of oxygen through the umbilical artery.

Results from this study are not showing that the estrogen receptor in the ovine fetal hypothalamus behaves biochemically in a manner different from the estrogen receptor of other species or at another time during the life. Rather, the present results suggest that both ICI 182,780 and estradiol activate a separate pathway that is not mediated by either ESR1 or ESR2. Results obtained after testing the differential expression of genes in the estrogen signaling pathway network (Fig. 3) suggest that in the fetal brain, binding of ICI 182,780 or estradiol to the membrane EGFR activates similar downstream signaling cascade. Also, it is possible that both estradiol and ICI 182,780 bind to GPR30 (GPER), a transmembrane G‐protein‐coupled receptor that has been shown to transactivate EGFR (Filardo 2002; Ge et al. 2012). ICI 182,780 is known to bind this receptor with high affinity (Thomas et al. 2005), and the transcriptomic responses to both ICI 182,780 and estradiol are consistent with activation of downstream pathways activated by GPR30. The mRNA expressions of GPR30 in the ovine fetal hypothalamus from 80 days of gestation to 1 week of extra‐uterine life were measured through qRT‐PCR (Fig. S2C). We found that the expression of GPR30 is significantly increasing at 130 days of gestation, to decrease before birth. However, mRNA levels increase again at 1 day of life but strongly decrease at 1 week.

**Figure 4:**
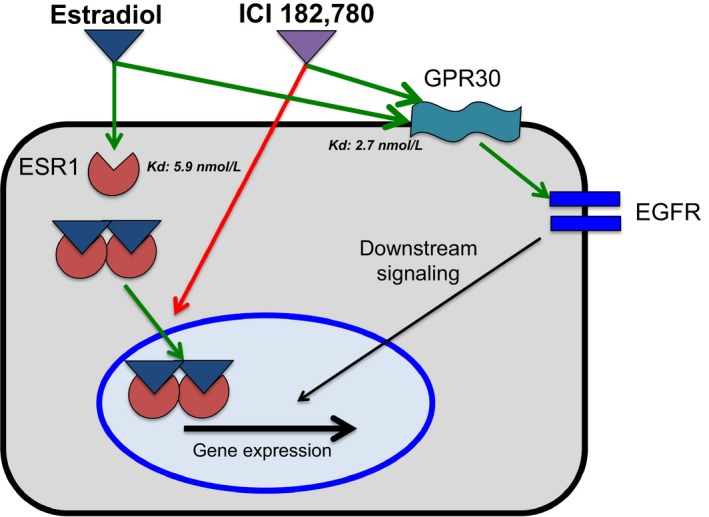
Diagram of Estradiol action on the fetal brain. Estradiol binds estrogen receptor alpha (ESR1), inducing its dimerization and nuclear translocation, which can be blocked by ICI 182,780. However, Estradiol and ICI 182,780 bind with high affinity, a transmembrane G‐protein‐coupled receptor (GPR30) which in turn could transactivate epidermal growth factor receptor (EGFR). Green arrow: stimulation, red arrow: inhibition.

The action of estradiol and ICI 182,780 on ESR1 or GPR30 may depend upon the abundance of these receptors in the developing brain, since estradiol has higher affinity for GPR30 (Kd: 2.7 nmol/L (Thomas et al. 2005)) than for ESR1 (Kd: 5.9 nmol/L (Weichman and Notides 1980)). Interestingly, mRNA levels of GPR30 in the ovine hypothalamus are significantly higher than mRNA levels of ESR1 at all ages from 80 days of fetal life to 1 week of extra‐uterine life, as measured through qRT‐PCR (Fig. S3). This difference ranged from 2.3 folds at 1 week of life to seven folds higher at 120–130 days of fetal life, coincident with the gestational ages when surgeries were performed. Thus, we can speculate that the main actions of estradiol and ICI 182,780 on the hypothalamic transcriptomics would be through the binding to GPR30 and transactivation of EGFR, inducing a parallel downstream signaling and consequently, a similar transcriptmic response (Fig. 4). Further studies could elucidate the roles of GPR30 in the developing fetal brain.

## Conclusion

We conclude that the transcriptomic response to ICI 182,780 in the fetal hypothalamus is similar to that of estradiol, and that ICI 182,780 does not block the actions of estradiol in this fetal brain region. Our findings suggest the possibility that the major action of estradiol on the fetal hypothalamus may not involve the estrogen receptor, but rather involve other receptor systems, such as GPR30. This is an intriguing idea that could inform a better understanding of estrogenization of the developing brain or of estrogen modulation in the endocrinology of the late gestation fetus.

## Conflict of Interest

No conflicts of interest, financial or otherwise, are declared by the author(s).

## Supporting information




**Figure S1.** Ontogeny of mRNA expression for (A) estrogen receptor alpha (ESR1) and (B) estrogen receptor alpha beta (ESR2) in the ovine fetal hypothalamus from 80 days of gestation to 1 day of extrauterine life (EUL). Expression levels were measured through microarray technology using an Agilent platform. Data are fold differences relative to mean expression at 80 days. a: different from 80 days values; b: different from 100 days values; c: different from 120 days values; d: different from 130 days values; e: different from 145 days values. For all statistical comparisons, *P* < 0.05 was used as the criterion for significance.

**Figure S2.** Ontogeny of mRNA expression for (A) estrogen receptor alpha (ESR1); (B) estrogen receptor beta (ESR2) and (C) G‐protein‐coupled estrogen receptor 1 (GPR30) in the ovine fetal hypothalamus from 80 days (d) of gestation to 1 week of extrauterine life (EUL). Expression levels were measured by qRT‐PCR. Data are fold differences relative to mean expression at 80 days. a: different from 80 days values; b: different from 100 day values; c: different from 120 day values; d: different from 130 days values; e: different from 145 days values. For all statistical comparisons, *P* < 0.05 was used as the criterion for significance.

**Figure S3.** mRNA expression of to G‐protein‐coupled estrogen receptor 1 (GPR30) and estrogen receptor beta (ESR2) relative to estrogen receptor alpha (ESR1) in the ovine fetal hypothalamus from 80 days of gestation to 1 week of extrauterine life (EUL). Expression levels were measured by qRT‐PCR. Grey Bars: ESR1, black bars: GPR30, white bars: ESR2. (*) different from ESR1 values. For all statistical comparisons, *P* < 0.05 was used as the criterion for significance.
