# Supplementary figures and images for: A transcriptomics model of estrogen action in the ovine fetal hypothalamus: evidence for estrogenic effects of ICI 182,780
Source: Physiol Rep. 2018 Sep 16;6(18):e13871. doi: 10.14814/phy2.13871 (PMC6139289; doi:10.14814/phy2.13871)

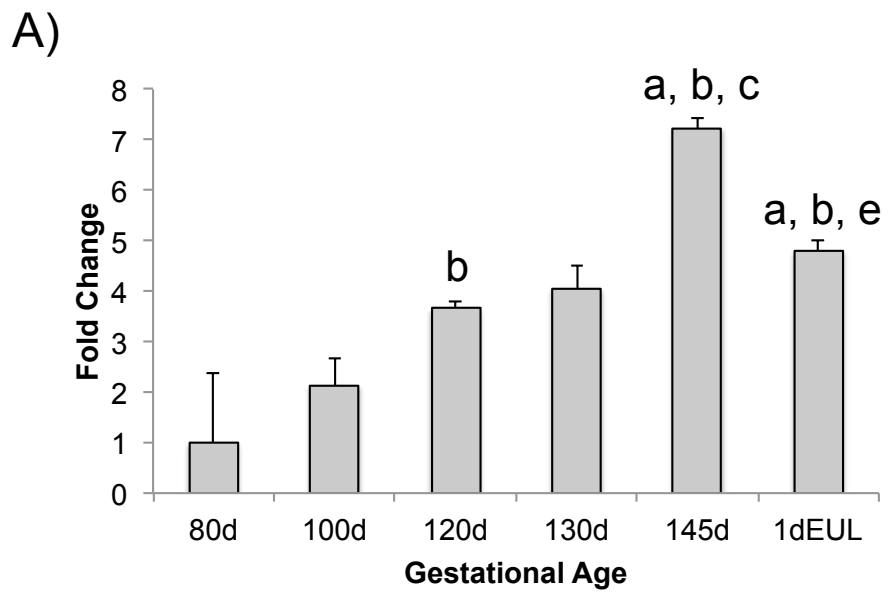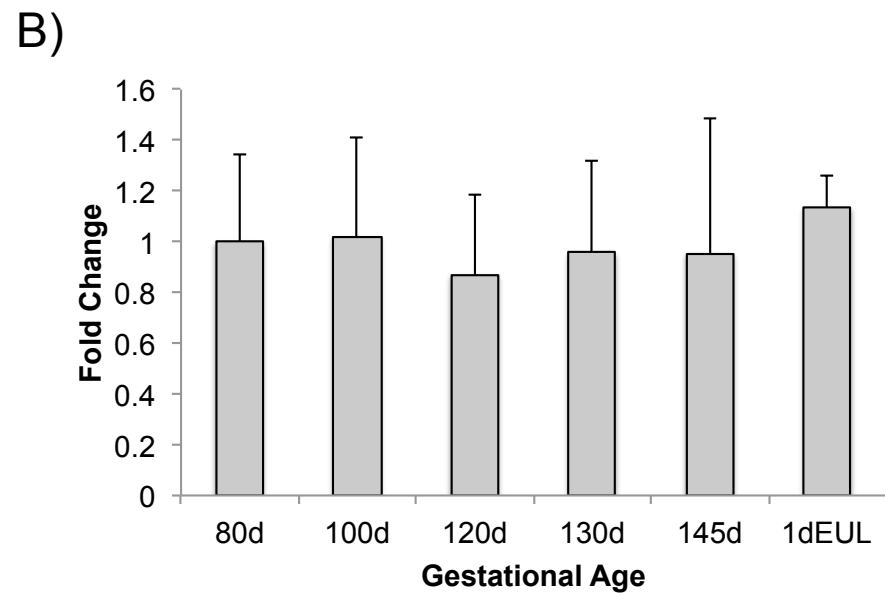

Supplement: Supplementary file 1 — Figure S1. Ontogeny of mRNA expression for (A) estrogen receptor alpha (ESR1) and (B) estrogen receptor alpha beta (ESR2) in the ovine fetal hypothalamus from 80 days of gestation to 1 day of extrauterine life (EUL). Expression levels were measured through microarray technology using an Agilent platform. Data are fold differences relative to mean expression at 80 days. a: different from 80 days values; b: different from 100 days values; c: different from 120 days values; d: different from 130 days values; e: different from 145 days values. For all statistical comparisons, P < 0.05 was used as the criterion for significance. [file PHY2-6-e13871-s001.pdf]

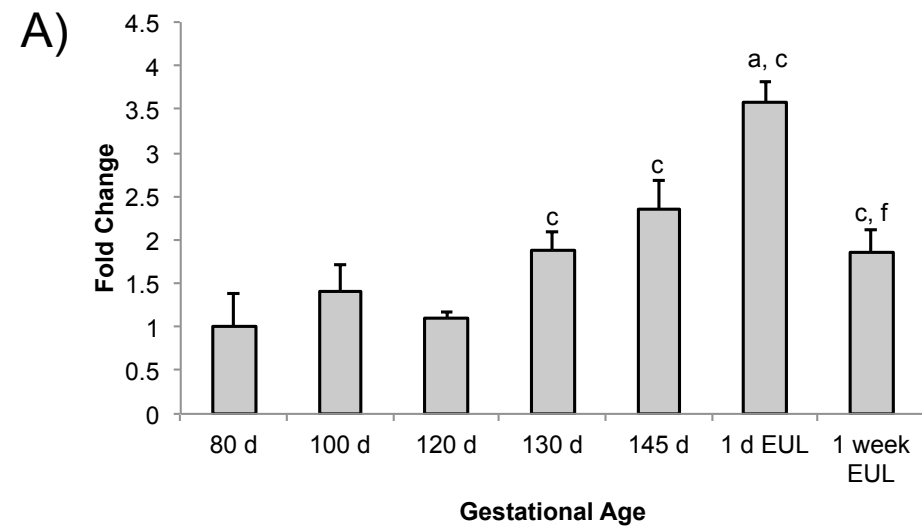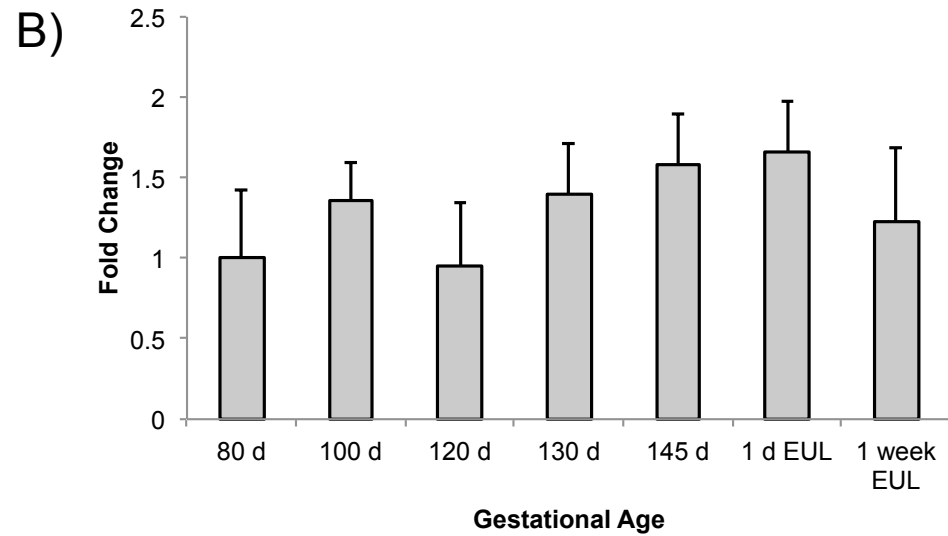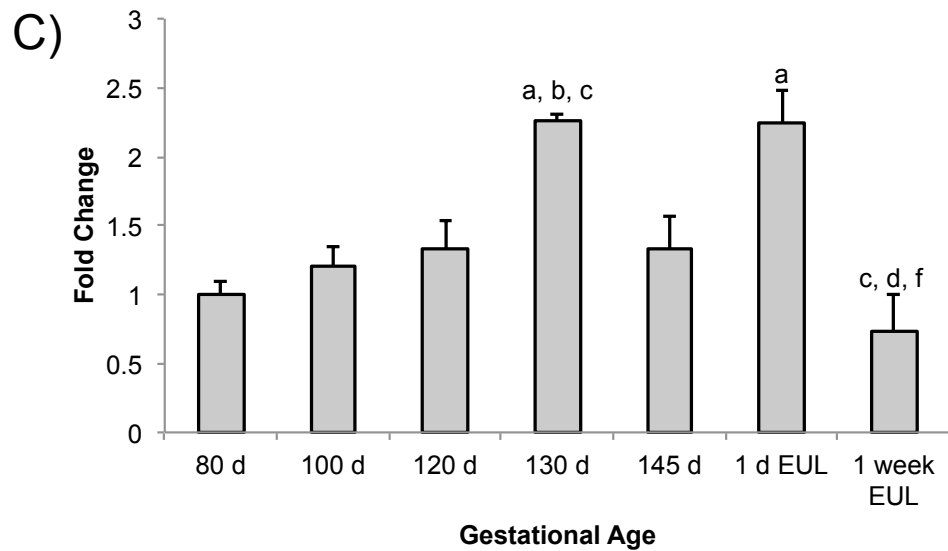

Supplement: Supplementary file 2 — Figure S2. Ontogeny of mRNA expression for (A) estrogen receptor alpha (ESR1); (B) estrogen receptor beta (ESR2) and (C) G‐protein‐coupled estrogen receptor 1 (GPR30) in the ovine fetal hypothalamus from 80 days (d) of gestation to 1 week of extrauterine life (EUL). Expression levels were measured by qRT‐PCR. Data are fold differences relative to mean expression at 80 days. a: different from 80 days values; b: different from 100 day values; c: different from 120 day values; d: different from 130 days values; e: different from 145 days values. For all statistical comparisons, P < 0.05 was used as the criterion for significance. [file PHY2-6-e13871-s002.pdf]

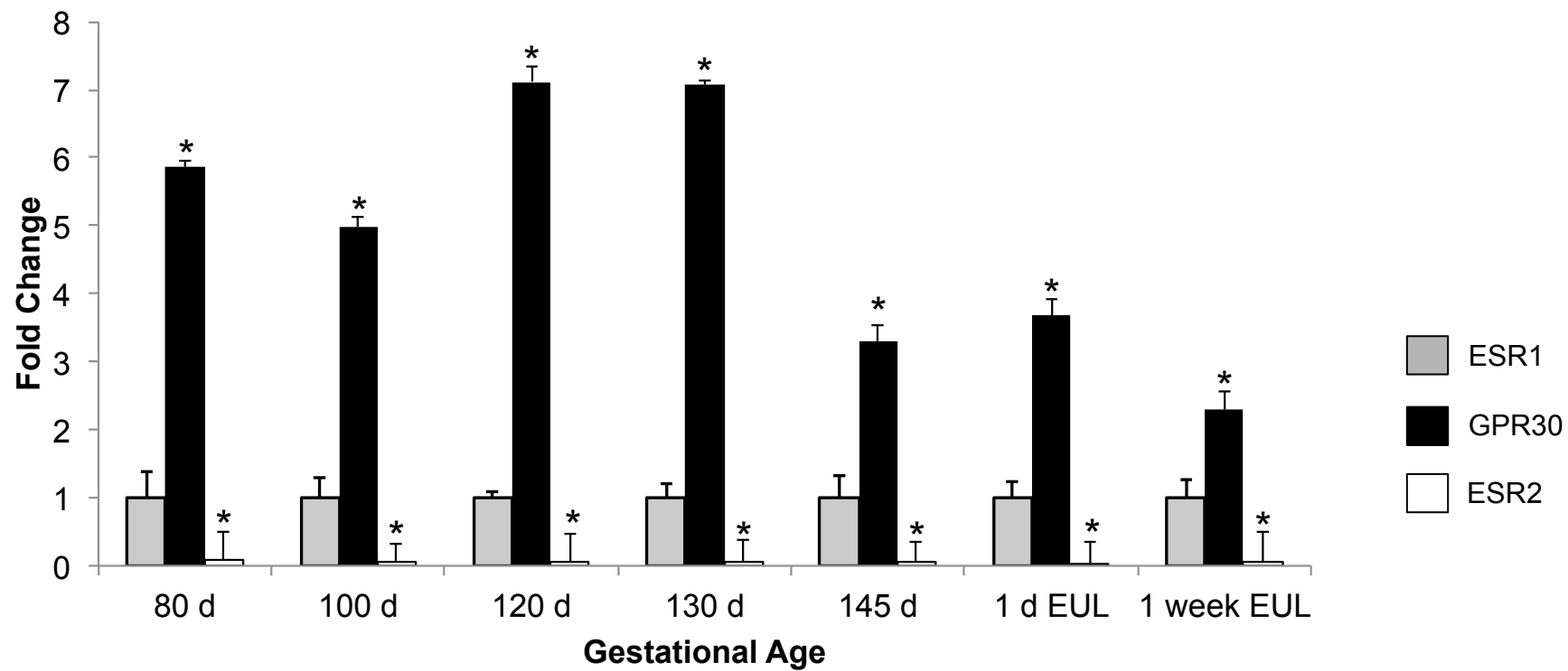

Supplement: Supplementary file 3 — Figure S3. mRNA expression of to G‐protein‐coupled estrogen receptor 1 (GPR30) and estrogen receptor beta (ESR2) relative to estrogen receptor alpha (ESR1) in the ovine fetal hypothalamus from 80 days of gestation to 1 week of extrauterine life (EUL). Expression levels were measured by qRT‐PCR. Grey Bars: ESR1, black bars: GPR30, white bars: ESR2. (*) different from ESR1 values. For all statistical comparisons, P < 0.05 was used as the criterion for significance. [file PHY2-6-e13871-s003.pdf]
